# Retinal Oxygenation in Inherited Diseases of the Retina

**DOI:** 10.3390/genes12020272

**Published:** 2021-02-14

**Authors:** Cengiz Türksever, Lisette T. López Torres, Christophe Valmaggia, Margarita G. Todorova

**Affiliations:** 1 VISTA Clinic Binningen BL, 4201 Binningen, Switzerland; med.c.turksever@hotmail.com; 2 Department of Ophthalmology, University of Basel, 4056 Basel, Switzerland; DRALISETTELOPEZ@hotmail.com (L.T.L.T.); christophe.valmaggia@kssg.ch (C.V.); 3 Department of Ophthalmology, Cantonal Hospital, 9007 St. Gallen, Switzerland; 4 Department of Ophthalmology, University of Zürich, 8091 Zürich, Switzerland

**Keywords:** retinal vessel oxygen saturation, oxygen exposure, inherited retinal diseases, metabolism–structure relationship

## Abstract

(1) Background: The aim of our study was to investigate the relationship between retinal metabolic alterations (retinal vessel oximetry, RO) and structural findings (retinal vessel diameter, central retinal thickness and retinal nerve fiber layer thickness, RNFL) in patients with inherited retinal diseases (IRDs). (2) Methods: A total of 181 eyes of 92 subjects were examined: 121 eyes of 62 patients with IRDs were compared to 60 eyes of 30 healthy age-matched controls. The retinal vessel oximetry was performed with the oxygen saturation measurement tool of the Retinal Vessel Analyser (RVA; IMEDOS Systems UG, Jena, Germany). The oxygen saturation in all four major peripapillary retinal arterioles (A-SO_2_; %) and venules (V-SO_2_; %) were measured and their difference (A-V SO_2_; %) was calculated. Additionally, retinal vessel diameters of the corresponding arterioles (D-A; µm) and venules (D-V; µm) were determined. The peripapillary central retinal thickness and the RNFL thickness were measured using spectral domain optical coherence tomography (SD-OCT) (Carl Zeiss Meditec, Dublin, CA, USA). Moreover, we calculated the mean central retinal oxygen exposure (cO_2_-E; %/µm) and the mean peripapillary oxygen exposure (pO_2_-E; %/µm) per micron of central retinal thickness and nerve fiber layer thickness by dividing the mean central retinal thickness (CRT) and the RNFL thickness with the mean A-V SO_2_. (3) Results: Rod-cone dystrophy patients had the highest V-SO_2_ and A-SO_2_, the lowest A-V SO_2_, the narrowest D-A and D-V and the thickest RNFL, when compared not only to controls (*p* ≤ 0.040), but also to patients with other IRDs. Furthermore, in rod-cone dystrophies the cO_2_-E and the pO_2_-E were higher in comparison to controls and to patients with other IRDs (*p* ≤ 0.005). Cone-rod dystrophy patients had the lowest cO_2_-E compared to controls and patients with other IRDs (*p* ≤ 0.035). Evaluated in central zones, the cO_2_-E was significantly different when comparing cone-rod dystrophy (CRD) against rod-cone dystrophy (RCD) patients in all zones (*p* < 0.001), whereas compared with controls and patients with inherited macular dystrophy this was observed only in zones 1 and 2 (*p* ≤ 0.018). The oxygen exposure was also the highest in the RCD group for both the nasal and the temporal peripapillary area, among all the evaluated groups (*p* ≤ 0.025). (4) Conclusions: The presented metabolic-structural approach enhances our understanding of inherited photoreceptor degenerations. Clearly demonstrated through the O_2_-E comparisons, the central and the peripapillary retina in rod-cone dystrophy eyes consume less oxygen than the control-eyes and eyes with other IRDs. Rod-cone dystrophy eyes seem to be proportionally more exposed to oxygen, the later presumably leading to more pronounced oxidative damage-related remodeling.

## 1. Introduction

Inherited retinal diseases (IRDs) have been summarized in a heterogeneous group presenting progressive photoreceptor degeneration [1,2,3,4]. Although the classical phenotypes of these entities are morphologically different [3,4], in severe phenotypes they may show similar generalized rod-cone or cone-rod dysfunction as measured by full field electroretinography (ffERG) [5,6,7,8,9]. The underlying photoreceptor dysfunction has been discussed to have a significant impact on the metabolic environment and might therefore be associated with remodeling and apoptosis that occur with the progression of IRDs [10,11,12,13,14,15,16,17].

Oxygen constitutes a crucial energy release reagent, the primarily biological tissue oxidant [18]. Compared with the human brain, the retina is known to share the most extensive metabolic supply and exchange in the body [19]. Oxygenation of the human retina is a dynamic process of which the regulation is maintained by different mechanisms. For the retina, not only lower oxygen levels, but also higher oxygen levels are destructive. Thus, in order to maintain proper visual function, adequate blood supply and oxygen metabolism are necessary [16,18,19,20]. Based on published data, the inner retina is supplied mainly by the retinal arterial vessels and the superficial retinal capillaries, whereas the outer retina is supplied generally by the choroidal vessels and to lesser extent by the deep retinal capillaries [20]. Notably, although the retinal blood flow is auto-regulated, the choroidal blood flow is not auto-regulated [20]. Therefore, in the presence of the degeneration of photoreceptor, the oxygen delivery to the outer retina remains unchanged, whereas the intraretinal oxygen level continues to increase [21,22,23,24].

According to the published research on microelectrode-based measurements in animal models, under normal conditions, regional variations in the intraretinal oxygen distribution have been found. Three oxygen consumption zones, corresponding to the superficial and deep retinal capillary and choroidal beds, have been clearly identified. According to these data, the metabolic supply of rod photoreceptors is supported mainly by the choroidal vessels and additionally by the deep retinal capillaries [25,26,27]. Under dark-adapted conditions, the metabolic activity of rod photoreceptors is further supported by oxygen diffusion from the superficial retinal capillary vessels [27,28,29].

In vivo studies on animal models of outer retinal degeneration have reported a marked reduction in oxygen utilization with acceleration of rod photoreceptor degeneration [26,27]. Although less oxygen is used by the degenerative photoreceptors, the oxygen delivery to the outer retina remains unchanged and the oxygenation of the inner retina increases [27]. With the progression of degeneration and continuous loss of photoreceptors, an increased oxygen level in the retina leads to the elevation of superoxide radicals and the generation of other reactive oxygen species in the retina, resulting in an increased risk of cell apoptosis [30]. Once the cones are involved, the central vision is impaired and is lost [31]. Subsequently, changes in the oxygen environment are reported to play a role in the progression of the degenerative process and the neurovascular remodeling [21,22,23,24].

Retinal vessel oximetry (RO) is a novel in vivo method, which allows researchers to study oxygen saturation of the retinal vessels and to explore metabolic alterations of the retina [19,32,33,34,35,36,37]. Recent RO studies on adults and children affected by inherited retinal disease have shown an altered oxygen metabolism by means of a significant increase in oxygen saturation in the retinal arterioles and venules, explained as a result of reduced oxygen consumption [38,39,40]. These findings were more pronounced in patients with rod-cone dystrophy (RCD) [38,39,40]. Furthermore, the oxygen saturation values correlated well with structural and functional changes [39,41,42,43]. In addition, peripapillary retinal vessel diameters were reduced proportionally to the functional and structural changes [39,44]. As the highest amount of oxygen is used by retinal photoreceptors, a reduction in retinal oxygen demand with a subsequent increase in oxygen saturation in the retinal vessels, followed by cellular apoptosis, has been hypothesized [39,41,43,45]. Based on the evidence discussed above, the degeneration of photoreceptors with secondary neurovascular remodeling seems to be a causative factor of increased retinal vessel oxygen saturation.

Spectral domain optical coherence tomography (SD-OCT) allows a precise in vivo evaluation of retinal structural alterations. Many contradictory results have been reported in regard to the thickness of the retinal nerve fiber layer (RNFL) in patients affected by IRD [39,46,47,48,49,50]. Nevertheless, the amount of peripapillary RNFL structural changes correlates well with the metabolic alterations detected by RO in IRDs [39]. In addition, the presence of cystoid macular edema (CME) in OCT imaging, reported to occur in 10%–50% of retinitis pigmentosa patients [51,52,53,54], has been well associated with the best-corrected visual acuity [55,56] and also with the degree of metabolic alterations and foveal vascular anomalies in RCD [42,57].

In order to deeper understand the compromised metabolic function and structural alterations in patients with IRDs, we continued studying the mechanism related to the retinal oxygen saturation which impacts the retinal structural alterations in different IRDs. Although the arterio-venous difference (A-V SO_2_; %), known to be proportional to the oxygen saturation, has been defined as a parameter for oxygen consumption [58], and the correlation between the level of structural damage and retinal vessel oxygen saturation has been confirmed in previous studies in RCD patients [39,41,42], the oxygen exposure for a certain amount of retinal and RNFL tissue (O_2_-E; %/µm) has not been studied yet. A central aim of the present study was therefore to calculate the mean retinal oxygen exposure per micron of central retinal thickness (CRT; µm) and per micron of nerve fiber layer thickness by dividing the mean CRT and RNFL thickness with the mean A-V SO_2_.

## 2. Materials and Methods

This cross-sectional consecutive study was performed on 92 subjects (53♀, 39♂). A total of 60 eyes of 30 healthy subjects (21♀, 9♂; mean 46.34 ± 10.50 years) were compared with 69 eyes affected by rod-cone dystrophy (RCD, 18 ♀ 17 ♂; 43.88 ± 13.54 years), 26 eyes with cone-rod dystrophy (CRD, 8♀ and 6 ♂; 41.08 ± 13.02 years) and 26 eyes with inherited macular dystrophy (IMD, 6 ♀ 7 ♂; 54.16 ± 13.49 years). The RCD subgroup included patients with a clinical picture of retinitis pigmentosa with predominantly rod over cone dysfunction. The CRD subgroup consisted of patients suffering from CRD with predominantly cone over rod dysfunction. The IMD subgroup included patients affected by Stargardt’s disease and Best’s disease.

Our study adhered to the tenets of the Declaration of Helsinki. All subjects provided informed consent before the study. The data were collected between September 2016 and December 2017.

### 2.1. Subjects

Our inclusion criteria for patients with IRDs were as follows: characteristic clinical and funduscopic features of IRD [3,4], Caucasian origin and typical electrophysiological findings [5,6,7,8,9]. The inclusion criteria for controls were as follows: Caucasian origin and having best-corrected visual acuity >0.8. Exclusion criteria for patients and controls were the presence of ocular and/or systemic pathology other than IRD (for instance, diabetes mellitus, hypertension or other metabolic and neurodegenerative diseases) and RO images with inadequate quality. Neither controls, nor patients with IRDs were under topical or systemic treatment with antioxidative, capillaroprotective, anti-inflammatory or antithrombotic action, which may influence the RO imaging. We performed for all controls and patients with IRD a standard ophthalmologic examination, including best-corrected visual acuity (Snellen charts), Goldmann applanation tonometry, biomicroscopy and fundoscopy.

### 2.2. Retinal Vessel Imaging

**Figure 1:**
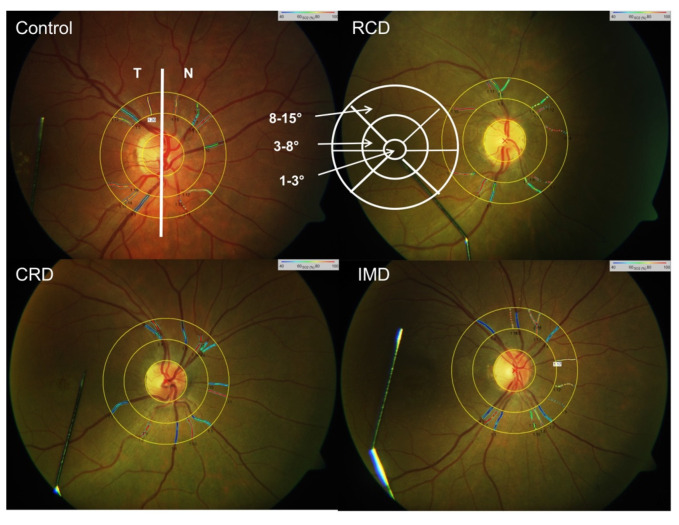
Vessel map of the retinal oximetry (RO) image, showing color-coded oxygen saturation (SO_2_) values of retinal vessels within the peripapillary annulus of a control. Scheme: 1.0–1.5 optic disc diameter distances from the optic disc margin. CRD—cone-rod dystrophy; IMD—inherited macular dystrophy; RCD—rod-cone dystrophy.

Each subject received tropicamide 0.5% and phenylephrine 1% eye drops for both eyes for mydriasis. The pupils were dilated to 7.0–8.0 mm. After a minimum of 20 min, four test–retest fundus images were obtained, as described previously [36]. We followed a standard procedure for RO acquisition in our clinic. Optic disc-centered fundus images, with a 50° field, were taken for each eye using the Retinal Vessel Analyser (RVA; Imedos UG, Jena, Germany), which was connected to the fundus camera FF450 (Carl Zeiss Meditec, Jena, Germany). Images of both eyes were obtained, starting with the right eye. At least four RO images with good image quality were taken. The three-channel luminance histogram tool of the RVA (Imedos UG) was used to control RO images for optimal brightness to reduce the possible effect of pigmentation of the retina and brightness on RO. As previously described, we selected for further analyses only images with optimal illumination, red channel illumination <160 step of the scale and green channel illumination >60 step of the scale [36]. An optic disc-centered image protocol was applied, where two rings, with a radius of 1.0 and 1.5 optic disc diameters, in the peripapillary area were plotted (Figure 1). The annulus between these two rings defined the area of interest in which we performed RO and vessel diameter measurements. All main arterioles and venules within the measurement area were analyzed. The average arteriolar and venular S-O_2_ (A-SO_2_ and V-SO_2_, %) and mean arteriolar and venular vessel diameter (D-A and D-V, µm) were obtained by simultaneously selecting the main vessels in all four quadrants. Their difference (A-V SO_2_, %), known to be proportional to the oxygen saturation of the retina, was calculated as well.

We evaluated the average, the naso-temporal, as well as the central SO_2_ parameters (A-SO_2_, V-SO_2_ and A-V SO_2_), retinal vessel diameters (D-A and D-V), as well as the corresponding oxygen exposure parameters (cO_2_-E and pO_2_-E). The naso-temporal values were calculated from the values corresponding to the nasal and temporal main peripapillary vessels. The ETDRS chart for calculation of the central retinal exposure parameters in zones was plotted around the fovea. 

### 2.3. Optical Coherence Tomography Imaging

**Figure 2:**
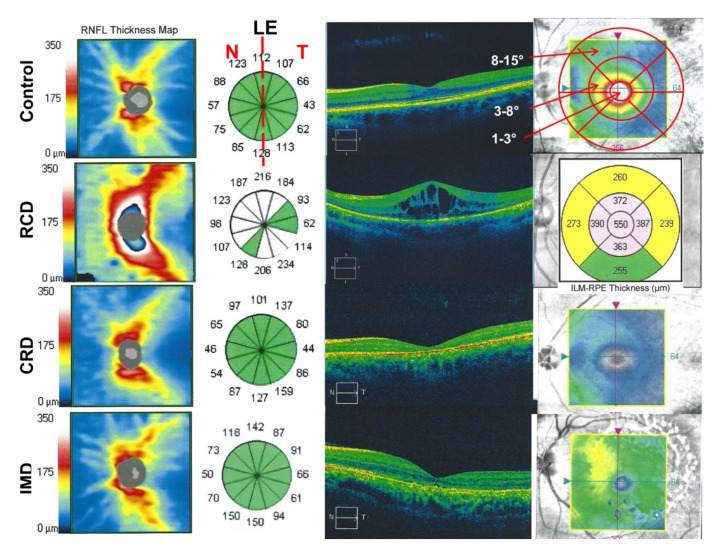
An example of RNFL thickness (12 scans, 6-mm optic nerve head-centered raster) and macular thickness protocol (Macular Cube 512 × 128) of a control subject (top, left eye), a patient with RCD (left eye), a patient with CRD (left eye) and a patient with IMD (bottom, left eye). The naso-temporal values were calculated from the values corresponding to the nasal and temporal main peripapillary vessels. The ETDRS chart for calculation of the central retinal exposure parameters in zones is plotted around the fovea.

For the evaluation of the retinal structure, we performed standard spectral domain OCT (SD-OCT) using Cirrus OCT (Carl Zeiss Meditec, Dublin, CA, USA). The OCT images were taken by implementing a macular thickness protocol (Macular Cube 512 × 128, Figure 1 and Figure 2) and a high-definition image-protocol (HD 5 Line Raster). The software of the Cirrus OCT provided a macular thickness map divided into nine subfields. For statistical analyses, the data were averaged, based on the anatomical and physiological structure of the central retina. We computed the mean macular thickness (µm) into three areas, as follows: zone 1 at 3°; zone 2 between 3° and 8°, and zone 3 between 8° and 15° (Figure 1 and Figure 2).

To evaluate the RNFL thickness, we performed an image protocol with a series of 12 scans, 6-mm optic nerve head-cantered raster, each on the Cirrus OCT (Carl Zeiss Meditec, Dublin, CA, USA). The average RNFL thickness (µm) was calculated automatically.

In addition, we calculated the mean retinal oxygen exposure (cO_2_-E; %/µm) and the mean peripapillary oxygen exposure (pO_2_-E; %/µm) per micron of central retinal thickness and per micron of nerve fiber layer thickness, by dividing the mean A-V SO_2_ by the mean CRT and by the mean RNFL thickness, respectively. The oxygen exposure in the central retina was assessed thereafter in zones, as follows: zone 1 (z1O_2_-E; %/µm); zone 2 (z2O_2_-C; %/µm), and zone 3 (z3O_2_-C; %/µm).

#### Statistical Procedures

For statistical analysis we used the IBM SPSS Statistics software, version 21 (International Business Machines Corp., Armonk, NY, USA). Mixed effects models are suitable for repeated measurements data. A linear mixed-effects model was performed for each pair of the tested methods, in which one parameter of the tested pair was a dependent variable. Results are presented as adjusted means and standard deviations for controls and the corresponding mean difference in patients’ subgroups with the respective *p*-values.

In the present study, ‘subject’ was taken as a random factor, and the ‘group’, ‘age’, ‘gender’, ‘location’ and the ‘eye’ were taken as fixed factors. The study groups were treated as covariates. The mean SO_2_ parameters (A-SO_2_, V-SO_2_, their difference: A-V SO_2_), oxygen exposure (O_2_-E), as well as the mean of the vessel diameter measurements (D-A and D-V) and the RNFL and macular thickness were taken as independent variables.

Our results are presented as *p*-values with corresponding regression coefficients. Statistical significance was defined as *p* < 0.05.

## 3. Results

In total, 181 eyes of 92 subjects were enrolled in the study: 69 eyes of 35 patients diagnosed with rod-cone dystrophy (RCD), 26 eyes of 13 patients with cone-rod dystrophy (CRD) and 26 eyes of 13 patients with inherited macular dystrophy (IMD) were compared to 60 eyes of 30 age-matched controls. All demographic characteristics of our participants are summarized in Table 1.

### 3.1. Increased A-SO_2_, V-SO_2_, O_2_-C and Decreased A-V SO_2_ Values in RCD Patients

**Figure 3:**
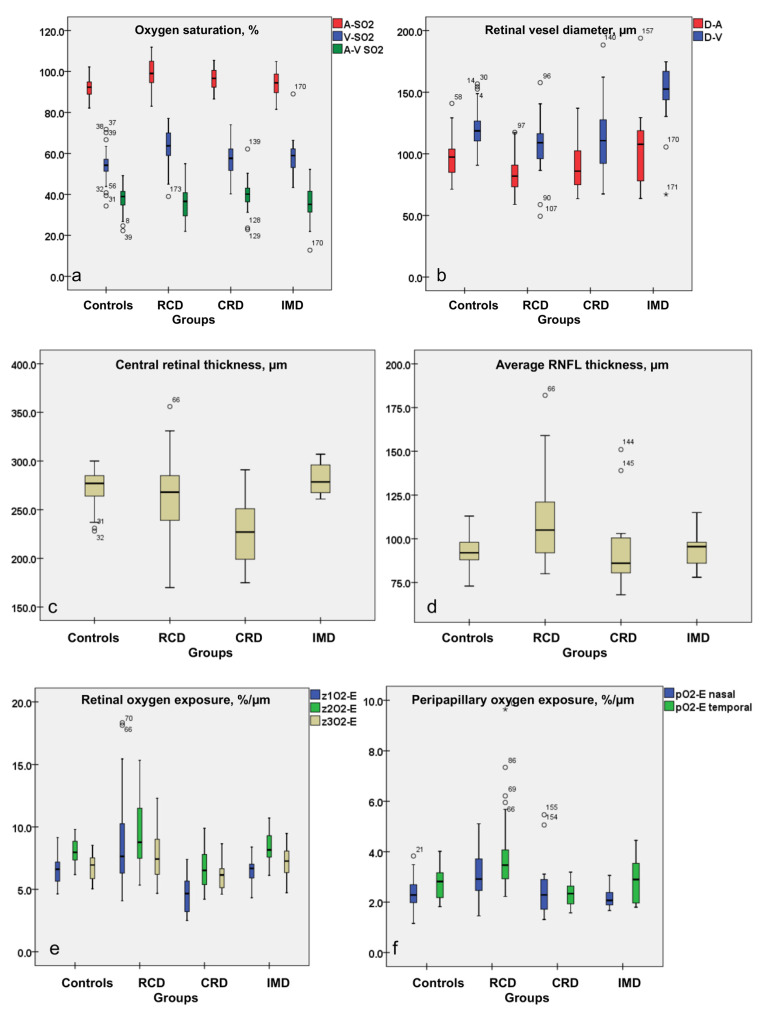
Box plots for the evaluated parameters, as follows: oxygen saturation (**a**); retinal vessel diameter (**b**), central retinal thickness (**c**), RNFL thickness (**d**), central oxygen exposure in zones (**e**) and peripapillary naso-temporal oxygen exposure (**f**) parameters.

In general, patients with RCD had higher average A-SO_2_ and V-SO_2_ and lower A-V SO_2_ values when compared to controls (*p* ≤ 0.04, ANOVA based on mixed-effect models; Figure 3a). For instance, in controls, the average A-SO_2_ and V-SO_2_ were measured at 92.08% and at 53.94%, respectively, and the corresponding average A-V SO_2_ at 38.21%. In RCD patients, the averaged retinal A-SO_2_ and V-SO_2_ showed a significantly increased difference (7.03% and 9.60%) when compared to controls, whereas the A-V SO_2_ decreased significantly by 2.98% (Table 2). The CRD group also revealed increased average A-SO_2_ values, and the IMD group presented increased average V-SO_2_ values compared to controls (*p* ≤ 0.012), but these were still not as high as those in the RCD group (Table 2, Figure 3a).

The box plots in Figure 3 represent the interquartile range; the short horizontal bold line depicts the median. In each graph the groups as labelled on the x-axis (from left to right: controls, rod-cone dystrophy (RCD), cone-rod dystrophy (CRD), inherited macular dystrophy (IMD)) and the evaluated parameters—on the y-axis.

### 3.2. Attenuated Retinal Vessel Diameters in RCD Patients

In general, the average peripapillary retinal vessel diameters, both the D-A and D-V, were significantly narrower in the RCD patients than in controls and in patients with IMD (*p* ≤ 0.001; Table 2, Figure 3b). Noticeably, the CRD group also showed narrower average peripapillary vessel diameters when compared to the IMD group (*p* ≤ 0.004), but not when compared to controls or RCD patients (Table 2, Figure 3b).

### 3.3. Peripapillary Retinal Nerve Fiber Layer Thickness Results: Thickest Peripapillary RNFL in RCD Patients

RCD patients have significantly greater average peripapillary RNFL thickness than controls and patients with other IRDs (*p* ≤ 0.001; Table 2, Figure 3d). In the temporal part of the RNFL this difference was more pronounced (*p* ≤ 0.001; Table 3, Figure 3f). Patients with IRDs other than RCD did not differ from controls in this respect.

### 3.4. Central Retinal Thickness in RCD

The OCT of the central retina imaging, consistent with previous studies on patients with IRDs [51,52,53,54,56,59], confirmed the loss of photoreceptors (IS/OS line integrity), distortions of the retinal microstructure and the presence of intra-retinal cystic spaces.

Within the entire IRD group only subjects with CRD differed from controls by means of significantly thinner central retina thickness with a mean difference from controls of 42.68 µm (*p* < 0.01; Table 2, Figure 3c). The mean CRT of CRD was significantly thinner in all zones, when evaluated in comparison to controls and patients with other IRDs (*p* ≤ 0.001) but to RCD only in zone 1 and zone 2 (*p* < 0.001; Table 4, Figure 3c).

The mean CRT of RCD did not differ significantly from controls (*p* = 0.227), probably due to the presence of intra-retinal cysts. However, evaluated in zones, the retinal thickness within zone 3 was significantly thinner than in controls (*p* = 0.043; Table 4).

### 3.5. Oxygen Exposure of the Retina in Patients with IRDs

Since the oxygen saturation, vessel diameters and central retinal and peripapillary structures are all affected in IRDs and to a greater extent in patients with RCD, we calculated the mean central retinal oxygen exposure (cO_2_-E; %/µm) and the mean peripapillary oxygen exposure (pO_2_-E; %/µm) per micron of central retinal thickness and per micron of RNFL thickness, by dividing the mean A-V SO_2_ with the mean CRT and respectively with the mean RNFL thickness.

In patients with RCD, the average central and peripapillary oxygen exposures (cO_2_-E and pO_2_-E) were significantly higher than those in controls, and were also higher than those in patients with other IRDs (*p* ≤ 0.005; Table 3 and Table 4). The oxygen exposure was also the highest in the RCD group for both the nasal and the temporal peripapillary area, among all the evaluated groups (*p* ≤ 0.025; Table 3). In addition, compared to the controls, for whom the peripapillary naso-temporal comparison showed significantly higher increased pO_2_-E values in the temporal area, this parameter did not reach statistically significant values in the RCD patients.

Cone-rod dystrophy patients had the lowest cO_2_-E compared to controls and patients with other IRDs (*p* ≤ 0.035; Table 2, Figure 3e). Evaluated in central zones, the cO_2_-E was significantly different comparing cone-rod dystrophy patients to rod-cone dystrophy patients in all zones (*p* < 0.001), whereas against controls and patients with inherited macular dystrophy only in zones 1 and 2 (*p* ≤ 0.018; Table 4, Figure 3e).

Patients with IMD did not differ from controls in these parameters.

## 4. Discussion

Alterations in the retinal structure and oxygen metabolism have been reported in patients with inherited retinal disease (IRD) and are mainly considered to be a consequence of metabolic and structural changes [38,39,46,47,54,60,61].

Deducting a structural–metabolic approach by evaluating patients with inherited retinal disease in comparison to controls, apart from studying retinal structure and oxygenation, we additionally evaluated the effect of retinal oxygen exposure for certain retinal- and RNFL tissues (O_2_-E). In order to do this, we calculated the mean retinal oxygen exposure per micron of central retinal thickness and per micron of nerve fiber layer thickness, O_2_-C (%/µm) by dividing the mean CRT and RNFL thickness with the mean A-V SO_2_.

### 4.1. Altered Structural and Metabolic Function in IRDs

Consistent with the results of the studies published to date [39,41,43], we reconfirmed increased A-SO_2_ and even more an increased V-SO_2_, with a corresponding decrease in the A-V SO_2_ values within the RCD group. A novel finding in the present study is that RCD patients also indicated significantly increased oxygen exposure when compared to controls and patients with other IRDs.

As rods are discussed to be much more oxygen demanding, their primary affliction would explain the more altered metabolic function when compared to other patients with IRD. This means that, following rod-cone dystrophy, due to the marked reduction in oxygen consumption under the continuing oxygen supply, more oxygen would be delivered to the inner retina, where the retinal oxygen saturation is measured. Increased intra-retinal levels of oxygen leads in turn to elevation of superoxide radicals and the generation of other reactive oxygen species in the retina, which increases the risk of cell apoptosis. The latter is clearly demonstrated in the presented data with much attenuated retinal vessels in the rod-cone dystrophy patients, and also with thickening of otherwise distorted central and peripapillary retina, consistent with the neurovascular remodeling that occurs with degeneration [21,22,23,24]. Here, increased oxygen flux due to the lack of the choroidal autoregulation [20] seems to have a significant impact on increased mean oxygen exposure in RCD patients, as confirmed now in our study.

The present study also revealed significantly altered central and peripapillary retinal structures that correspond to disturbed oxygenation, with the RCD group found to be the most affected among IRD patients.

### 4.2. Altered Central Structure and Metabolic Function in IRDs

The OCT imaging of the central retina, consistent with previous studies on patients with IRD [55,56,59], confirmed the loss of photoreceptors with IS/OS line integrity, distortions of the retinal microstructure and/or macular edema. Although the central retinal thickness in RCD patients did not differ from that of controls (*p* = 0.227), it was significantly thinner in CRD. The later held true when compared to controls and also to patients with other inherited retinal dystrophies (*p* < 0.001).

A novel finding in the present study is the significantly increased central oxygen exposure in our RCD group when compared to controls and patients with other IRDs (*p* ≤ 0.005). Interestingly, even if the central retinal thickness in RCD patients was within the normal range (*p* = 0.227), the oxygen exposure was significantly increased. This finding was more pronounced in zone 1 (up to 3°) and zone 2 (3°–8°), when patients with other IRDs were considered.

Many studies have already reported on structural and functional alterations within the RCD group in the macular area. Outside zone 2 (3°–8°), a study by Konieczka et al. found a reduction in mfERG responses corresponding to the reduction in the central retinal layer thickness [56]. Funatsu et al. [62] reported in RCD patients reduced central retinal sensitivity measured by microperimetry corresponding to reduced outer retinal thickness at 6°–8°. Several studies on fundus autofluorescense imaging in RCD patients have paid attention to increased annular hyperautofluorescence in the parafoveolar area (within 3.0° to 10.5°), which is supposed to indicate a metabolic abnormality [63,64]. Considerably, the progression of the hyperautofluorescence ring constriction has been found to correlate strongly with retinal eccentricity. More precisely, the mean outer ring (within 3.0° to 10.5°) has shown significantly rapid radius reduction per year, compared to the mean inner ring (0.2°–3.0°) [64]. The authors suggested that the rod system dysfunction in RCD patients may lead to a consequent cone dysfunction and thus to progressive visual field constriction and central vision impairment. In agreement with this, studies using adaptive optics scanning laser en-face images in RCD patients have shown reduced cone photoreceptor density and a strong correlation with retinal eccentricity [59,65]. Since using antioxidants in RCD models supposedly decreases the oxidative damage and prevents the death of cone photoreceptors, it is inferred that oxidative damage is a major contributor to cone photoreceptor apoptosis [66].

In agreement with the results of the studies stated above, in our RCD patients a significant oxygen exposure at the region corresponding to zone 2 was found. We suppose that as part of the tissue apoptosis, increased oxygen exposure occurs, with a consequent increase in the oxidative stress of rod photoreceptors on the border the retina, which is severely affected by remodeling. These results seem to have a significant impact on abnormal autofluorescence, reduced sensitivity in microperimetry and mfERG measurement, as well as on distortions of retinal microstructure in the central retina.

Contrarily, in our CRD patients, opposite to the controls and other IRD patients, the CRT was significantly reduced (*p* < 0.001), as well as the oxygen exposure (*p* ≤ 0.035). In accordance with the published studies, the cone density is higher in the central retina [67,68,69]. It is, however, also known that rods are more oxygen-demanding than cones [27,28,29]. Therefore, in the presence of cone-rod dystrophy, where cone photoreceptors are primarily affected and rod photoreceptors remain intact for a long period, an oxygen influx from choroidea into the retina is expected to remain unchanged, explaining our results.

### 4.3. Altered Peripapillary Metabolic Function in IRDs

Peripapillary RNFL thickness in RCD patients has gained attention in many studies and has been discussed as related to the neurovascular remodeling [39,46,47,48,49,50]. In our patient group, the RNFL was significantly thicker in the RCD group when compared to controls and to patients with other IRDs. Interestingly, in the temporal part of the RNFL this difference was more pronounced. Furthermore, in the peripapillary temporal retina, oxygen use as determined by the A-V SO_2_ parameter was significantly reduced compared to controls and cone-rod dystrophies (*p* ≤ 0.019). In the comparison with IMDs, this parameter was significantly altered only in the nasal RNFL (*p* = 0.004). The oxygen exposure was, however, the highest in the RCD group for both the nasal and the temporal peripapillary area, among all the evaluated groups. However, compared to controls, for whom the peripapillary naso-temporal difference showed significantly increased oxygen exposure values in the temporal area, the RCD patients did not reach statistically significant values in this parameter. A possible explanation for these results could be as follows.

According to the topographic mapping in controls, more rod photoreceptors would be observed in the temporal retina, whereas more cone photoreceptors would be observed in the nasal retina [69]. This finding has already been discussed as related to the increased intensity of the cellular metabolism in the macula in controls [70]. Thus, although the capillary-free zone close to the macula is supposed to have higher extracellular oxygen diffusion, the higher density of photoreceptors and ganglion cells in the macula yields to higher oxygen extraction in the temporal peripapillary retina. This would explain the higher oxygen exposure values in the temporal peripapillary area in controls, which we were able to document.

A novel finding in the present study is the loss of peripapillary naso-temporal difference in oxygen exposure values within the RCD group. Compared to controls and CRDs, this naso-temporal difference in the RCD group was not present. This finding could be explained by the more rapid and progressive degeneration measured in RCD in the temporal part of the retina, with a consecutively more increased choroidal flux, and correspondingly increased oxygen exposure, as confirmed now in the present study. Contrarily, any possible reduction in the peripapillary naso-temporal oxygen exposure difference in RCD may serve as a predictor for the progression of RCD once the cones, which are denser in the macula, are affected.

In eyes with cone-rod dystrophy, based on the underlined primary degeneration of cones and according to the topographic mapping of the cone density in controls, the oxygen exposure map would be presented differently. Here, due to the primary affliction of cone photoreceptors, and thus greater effects on the nasal retina, the oxygen exposure may increase in the peripapillary nasal retina, diminishing the naso-temporal difference compared to controls and patients with RCD. Furthermore, taking the generalized cone photoreceptor degeneration in patients with CRD into account, less oxygen influx from the choroidea in to the retina may be measured, resulting in less affected oxygen exposure in CRDs. The latter would explain why the RNFL thickness, as well as the diameters of the peripapillary vessels, remain stable for a longer period. This explanation may serve also to indicate that changes in cone photoreceptors’ oxygen metabolism are limited, as clearly demonstrated with less pronounced tissue remodeling.

Our study has, however, some limitations that include among others the genetic heterogeneity, different clinical stages of our IRD patients and the small sample size in the IMD subgroup. in addition, possible interactions between retinal blood flow, oxygen delivery, oxygen consumption, as well as changes in choroidal contribution should have been taken into consideration for the interpretation of oxygen extraction. Therefore, further studies are needed to evaluate to what extent the altered retinal and choroidal perfusion in IRD patients may contribute to the measured metabolic dysfunction.

## 5. Conclusions

Clearly demonstrated through the applied metabolic–structural approach, we were able to differentiate RCD patients not only from controls, but also from patients with other IRDs.

With the adopted pO_2_-E/cO_2_-E model, the oxidative stress in the retina may be accessed in vivo and be used as a metabolic parameter in understanding inherited retinal diseases. Furthermore, these parameters of mean oxygen exposure in relation to the amount of central retinal thickness and retinal nerve fiber layer thickness may serve as a biomarker in evaluating the progression rate of the degeneration, as well as the effect of future therapies.

## Figures and Tables

**Table 1:** Demographic characteristics including age, gender, best corrected visual acuity (BCVA, Snellen charts) in eyes of controls and patients suffering inherited retinal diseases in subgroups as mean ± standard deviation (SD).

Groups	Number of Subjects	Evaluated Eyes		Age, Y; Mean (±SD)	Gender (♀:♂)	Mean BCVA, (±SD) (Snellen Charts)
		RE	LE			
Controls	30	30	30	46.34 (10.50)	21:9	1.0 (0.03)
RCD	35	34	35	43.88 (13.54)	18:17	0.53 (0.26)
CRD	13	13	13	41.08 (13.02)	8:6	0.47 (0.36)
IMD	13	13	13	54.16 (13.49)	6:7	0.75 (0.30)

**Table 2:** Oxygen saturation parameters (A-SO_2_, V-SO_2_ and A-V SO_2_), retinal vessel diameters (D-A and D-V), the central retinal thickness and the RNFL thickness and the oxygen exposure (O_2_-E).

Adjusted Means in Groups (±SD)				Comparison between Groups		ANOVA, Based on Mixed Effects Model	
Controls	RCD	CRD	IMD	Group 1	Group 2	Mean Difference	*p*-Values between Groups
A-SO_2_ (%)							
92.08 (4.51)	99.12 (6.42)	96.55 (4.95)	94.30 (6.03)	Controls Controls Controls RCD RCD CRD	RCD CRD IMD CRD IMD IMD	−7.03 −4.47 −2.21 2.57 4.82 −2.26	<**0.001** **0.001** 0.102 0.052 <**0.001** 0.148
V-SO_2_ (%)							
53.94 (6.65)	63.55 (7.64)	56.67 (8.03)	58.61 (8.63)	Controls Controls Controls RCD RCD CRD	RCD CRD IMD CRD IMD IMD	−9.60 −2.72 −4.66 6.88 4.94 −1.94	<**0.001** 0.138 **0.012** <**0.001** **0.006** 0.358
A-V SO_2_ (%)							
38.21 (5.52)	35.54 (8.59)	39.86 (7.74)	35.69 (9.00)	Controls Controls Controls RCD RCD CRD	RCD CRD IMD CRD IMD IMD	2.98 −1.65 2.52 −4.32 −0.15 4.17	**0.040** 0.374 0.177 **0.018** 0.935 0.053
D-A (μm)							
96.21 (13.910)	84.12 (14.43)	91.20 (19.63)	106.93 (29.65)	Controls Controls Controls RCD RCD CRD	RCD CRD IMD CRD IMD IMD	12.10 5.01 −10.72 −7.09 −22.82 −15.71	<**0.001** 0.236 **0.027** 0.086 <**0.001** **0.004**
D-V (μm)							
121.00 (14.67)	107.60 (17.88)	112.23 (28.58)	147.70 (26.31)	Controls Controls Controls RCD RCD CRD	RCD CRD IMD CRD IMD IMD	13.40 8.77 −26.70 −4.63 −40.10 −35.47	**0.001** 0.074 <**0.001** 0.329 <**0.001** <**0.001**
Central retinal thickness (μm)							
271.67 (19.60)	263.80 (36.02)	229.00 (35.84)	281.88 (15.77)	Controls Controls Controls RCD RCD CRD	RCD CRD IMD CRD IMD IMD	7.87 42.68 −10.20 34.80 −18.07 −52.88	0.227 <**0.001** 0.275 <**0.001** **0.037** <**0.001**
RNFL thickness (μm)							
92.83 (8.42)	109.85 (22.91)	92.50 (20.63)	92.89 (9.90)	Controls Controls Controls RCD RCD CRD	RCD CRD IMD CRD IMD IMD	−17.02 0.33 −0.06 17.35 16.96 −0.39	<**0.001** 0.946 0.991 <**0.001** **0.001** 0.947
cO_2_-E (µm/%)							
6.92 (1.05)	8.08 (2.15)	5.88 (1.30)	7.51 (1.14)	Controls Controls Controls RCD RCD CRD	RCD CRD IMD CRD IMD IMD	−1.16 1.05 −0.60 2.20 0.56 −1.64	**0.002** **0.035** 0.242 <**0.001** **0.005** **0.005**
pO_2_-E (µm/%)							
2.35 (0.36)	3.36 (1.02)	2.36 (0.60)	2.58 (0.64)	Controls Controls Controls RCD RCD CRD	RCD CRD IMD CRD IMD IMD	−1.01 −0.01 −0.23 1.00 0.78 −0.21	<**0.001** 0.946 0.320 <**0.001** <**0.001** 0.404

**Table 3:** The naso-temporal RNFL thickness parameters and the corresponding oxygen exposure parameters (pO_2_-E nasal, pO_2_-E temporal).

Adjusted Means in Groups (±SD)				Comparison between Groups		ANOVA, Based on Mixed Effects Model	
Controls	RCD	CRD	IMD	Group 1	Group 2	Mean Difference	*p*-Values between Groups
RNFL nasal (μm)							
87.80 (13.97)	103.88 (28.02)	90.10 (31.83)	88.62 (13.10)	Controls Controls Controls RCD RCD CRD	RCD CRD IMD CRD IMD IMD	−16.07 −4.30 −0.82 11.80 15.25 3.48	**0.001** 0.490 0.904 0.053 **0.023** 0.649
RNFL temporal (μm)							
96.23 (11.91)	113.97 (25.35)	88.36 (17.16)	96.88 (14.03)	Controls Controls Controls RCD RCD CRD	RCD CRD IMD CRD IMD IMD	−17.740 7.874 −0.650 25.614 17.090 −8.524	<**0.001** 0.121 0.904 <**0.001** **0.001** 0.164
A-V SO_2_ nasal (%)							
38.20 (5.36)	37.04 (5.89)	36.88 (5.30)	41.62 (6.91)	Controls Controls Controls RCD RCD CRD	RCD CRD IMD CRD IMD IMD	1.151 1.313 −3.421 0.162 −4.572 −4.734	0.390 0.393 **0.039** 0.912 **0.004** **0.008**
A-V SO_2_ temporal (%)							
36.35 (6.45)	32.60 (6.71)	38.39 (6.63)	34.29 (8.61)	Controls Controls Controls RCD RCD CRD	RCD CRD IMD CRD IMD IMD	3.743 −2.041 2.058 −5.784 −1.685 −4.099	**0.019** 0.261 0.298 **0.001** 0.380 0.055
pO_2_-E nasal (µm/%)							
2.35 (0.58)	3.03 (0.91)	2.54 (1.05)	2.15 (0.42)	Controls Controls Controls RCD RCD CRD	RCD CRD IMD CRD IMD IMD	−0.681 −0.184 0.200 0.498 0.881 −0.383	**0.001** 0.438 0.457 **0.025** **0.001** 0.177
pO_2_-E temporal (µm/%)							
2.73 (0.66)	3.82 (1.45)	2.31 (0.45)	2.83 (0.91)	Controls Controls Controls RCD RCD CRD	RCD CRD IMD CRD IMD IMD	−1.095 0.420 −0.104 1.516 0.991 0.525	<**0.001** 0.162 0.765 <**0.001** **0.004** 0.159

**Table 4:** The retinal thickness in zones and the corresponding oxygen exposure parameters (z1O_2_-E, z2O_2_-E and z3O_2_-E).

Adjusted Means in Groups (±SD)				Comparison between Groups		ANOVA, Based on Mixed Effects Model	
Controls	RCD	CRD	IMD	Group 1	Group 2	Mean Difference	*p*-Values between Groups
Retinal thickness, Zone 1 (μm)							
259.56 (28.32)	272.11 (77.02)	179.22 (43.32)	246.69 (15.92)	Controls Controls Controls RCD RCD CRD	RCD CRD IMD CRD IMD IMD	−13.74 80.34 12.87 94.08 26.61 −64.47	0.274 <**0.001** 0.473 <**0.001** 0.109 **0.001**
Retinal thickness, Zone 2 (μm)							
318.79 (12.34)	303.16 (55.52)	258.49 (44.85)	312.26 (27.29)	Controls Controls Controls RCD RCD CRD	RCD CRD IMD CRD IMD IMD	15.86 60.30 6.52 44.44 −9.34 −53.78	0.094 <**0.001** 0.628 <**0.001** 0.452 **0.001**
Retinal thickness, Zone 3 (μm)							
268.69 (25.47)	255.92 (33.22)	239.47 (30.44)	270.88 (27.36)	Controls Controls Controls RCD RCD CRD	RCD CRD IMD CRD IMD IMD	13.26 29.22 −2.18 15.96 −15.44 −31.40	**0.043** **0.001** 0.814 0.053 0.073 **0.003**
z1O_2_-E (µm/%)							
6.62 (1.18)	8.48 (3.06)	4.66 (1.52)	6.59 (1.05)	Controls Controls Controls RCD RCD CRD	RCD CRD IMD CRD IMD IMD	−1.86 1.96 0.03 3.82 1.90 −1.93	<**0.001** **0.004** 0.961 <**0.001** **0.004** **0.015**
z2O_2_-E (µm/%)							
8.11 (1.03)	9.36 (2.58)	6.67 (1.66)	8.30 (1.20)	Controls Controls Controls RCD RCD CRD	RCD CRD IMD CRD IMD IMD	−1.26 1.44 −0.19 2.70 1.07 −1.63	**0.004** **0.014** 0.755 <**0.001** 0.059 **0.018**
z3O_2_-E (µm/%)							
6.83 (1.03)	7.81 (1.02)	6.15 (1.20)	7.24 (1.36)	Controls Controls Controls RCD RCD CRD	RCD CRD IMD CRD IMD IMD	−0.98 0.68 −0.42 1.66 0.56 −1.10	**0.006** 0.150 0.399 <**0.001** 0.222 0.051

## Data Availability

Data Access and Responsibility: Margarita Todorova has full access to all the data in the study and hold complete responsibility for the data integrity and the accuracy of the analysis.
